# Copper, lead and zinc interactions during phytoextraction using *Acer platanoides* L.—a pot trial

**DOI:** 10.1007/s11356-022-23966-x

**Published:** 2022-11-15

**Authors:** Mirosław Mleczek, Anna Budka, Monika Gąsecka, Sylwia Budzyńska, Kinga Drzewiecka, Zuzanna Magdziak, Paweł Rutkowski, Piotr Goliński, Przemysław Niedzielski

**Affiliations:** 1 Department of Chemistry, Poznań University of Life Sciences, Wojska Polskiego 75, 60-625 Poznań, Poland; 2 Department of Mathematical and Statistical Methods, Poznań University of Life Sciences, Wojska Polskiego 28, 60-637 Poznań, Poland; 3 Department of Forest Sites and Ecology, Poznań University of Life Sciences, Wojska Polskiego 71F, 60-625 Poznań, Poland; 4 Faculty of Chemistry, Adam Mickiewicz University in Poznań, Uniwersytetu Poznańskiego 8, 61-614 Poznań, Poland

**Keywords:** Antagonism, Metals, Plant phenolics, Synergism, Trace elements, Trees

## Abstract

Of the many environmental factors that modulate the phytoextraction of elements, little has been learnt about the role of metal interactions. The study aimed to show how different concentrations of Cu, Pb and Zn in the cultivation medium influenced the biomass, plant development and phytoextraction abilities of *Acer platanoides* L. seedlings. Additionally, the impact on the content and distribution of Ca, K, Mg and Na in plant parts was studied with an analysis of phenols. Plants treated with a mixture of two metals were characterised by lower biomass of leaves and higher major elements content jointly than those grown in the salt of one element. Leaves of *A. platanoides* cultivated in Pb_5_ + Zn_1_, Pb_1_ + Zn_1_ and Pb_1_ + Zn_5_ experimental systems were characterised by specific browning of their edges. The obtained results suggest higher toxicity to leaves of Pb and Zn present simultaneously in Knop solution than Cu and Pb or Cu and Zn, irrespective of the mutual ratio of the concentrations of these elements. Antagonism of Cu and Zn concerning Pb was clearly shown in whole plant biomass when one of these elements was in higher concentration (5 mmol L^−1^) in solution. In the lowest concentrations (1 mmol L^−1^), there was a synergism between Cu and Zn in plant roots. Plants exposed to Zn_5_, Cu_1_ + Pb_5_, Pb_5_ + Zn_1_ and Cu_1_ + Zn_1_ were characterised by higher total phenolic content than the rest plants. Both the presence and the concentration of other elements in the soil are significant factors that modulate element uptake, total phenolic content, and plant development.

**Graphical Abstract:**

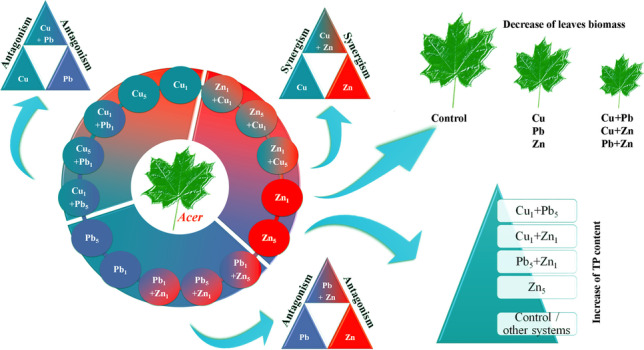

## Introduction

The effective phytoextraction of elements from polluted areas requires an appropriate selection of plants capable of adaptation and undisturbed growth (Chandra and Kumar 2017; Lai and Chen 2009). The vast majority of studies are focused on searching for the most effective plants that show high metal uptake rates and translocation to the above-ground parts, including transgenic ones (DalCorso et al. 2019; Suman et al. 2018). In this approach, the key factor stimulating the phytoextraction of elements is their increased transport through the plant, rather than the induction of intracellular ligands (phytochelatins and metallothioneins) to strengthen the metal complexation process (Krzesłowska 2011). As described in the literature, the introduction and overexpression of genes with an important role in the uptake of toxic elements, their translocation, and sequestration are the most common strategies to improve the phytoremediation potential of plants (Fasani et al. 2018; Yan et al. 2020).

For phytoremediation efficiency, in addition to the plant being an integral part of the process, environmental factors are crucial, especially the characteristics of the contaminated matrix (soil, post-mining wastes, sludges) (Magdziak et al. 2015; Sheoran et al. 2016). The concentration of highly toxic elements in the soil and its physico-chemical properties usually significantly modulate the plant response (Iqbal et al. 2014; Shehata et al. 2019). As commonly known, soil pH and organic matter content and water regime (with the following implication of osmotic disturbances in plants) are key factors influencing the uptake of elements by plants (Angle et al. 2003; Dolar and Keeney 1971; Nadjimi 2021; Rucińska-Sobkowiak 2016). Moreover, the salinity, organic amendments or microorganisms also affect plant performance during the phytoremediation process (DalCorso et al. 2019; Hasanuzzaman et al. 2014; Sabir et al. 2014). In recent years, particular attention has been paid to the plant microbiome as an essential factor regulating the uptake of heavy metals (Ma et al. 2016).

Among numerous factors influencing the transport of elements from the polluted substrate to the plant, relatively little attention has been paid to the interactions between elements (Ghori et al. 2015; Zaranyika and Nyati 2019). The main reasons for the limited number of studies of metal interactions are (i) complexity of factors in field studies forcing the need to conduct research under controlled conditions using artificially polluted substrates, and (ii) the difficulty in interpreting the results in the case of experimental systems consisting of more than 3 elements (Mleczek 2015). The evaluation of metal interactions in field experiments is hardly possible due to the multidimensional relations that simultaneously include numerous elements, including mineral nutrients and toxic elements (Ismael et al. 2019; Mleczek et al. 2011, 2012; Tangahu et al. 2011). Mutual interactions may influence the intracellular production of chelating agents and their exudation to the rhizosphere, e.g. low molecular weight organic acids, causing lower/higher phytoextraction of particular elements (Magdziak et al. 2011). Further, metal interactions may affect the induction of enzymatic and non-enzymatic antioxidants influencing metal toxicity (Israr et al. 2011). They may lead to nutrient deficiency due to the competitive uptake of toxic elements (Robson and Pitman 1983).

Copper (Cu), lead (Pb) and zinc (Zn) are widely distributed heavy metals present in the soil in concentrations ranging from ~ 4 to 2270, < 3 to 886 and 1 to 239 mg kg^−1^ for European topsoil samples, with a median concentration of ~ 12, 15 and 48 mg kg^−1^, respectively (Salminen et al. 2005). With the exception of cadmium (Cd) and arsenic (As), the above mentioned metals are the most frequently studied elements in phytoremediation aspects (Amin et al. 2021; Butkus and Baltrénaité-Gediene 2007; Lorenc-Plucińska et al. 2013; Molnárová et al. 2018; Napoli et al. 2019; Riza and Hoque 2021; Shin et al. 2012; Yanqun et al. 2004). Consequently, the general pattern of interactions between Cu, Zn, and Pb is well documented. However, the data for tree species are still limited (Adamczyk-Szabela et al. 2020; Israr et al. 2011; Luo and Rimmer 1995). Thus, this study aimed to compare the phytoextraction rates of Cu, Pb and Zn for Norway maple (*Acer platanoides* L.) exposed to single or simultaneous metal treatments with different concentration ratios along with biomass investigations and the assessment of total phenolics as a parameter of metal-induced stress.

## Materials and methods

### Plant material

**Fig. 1:**
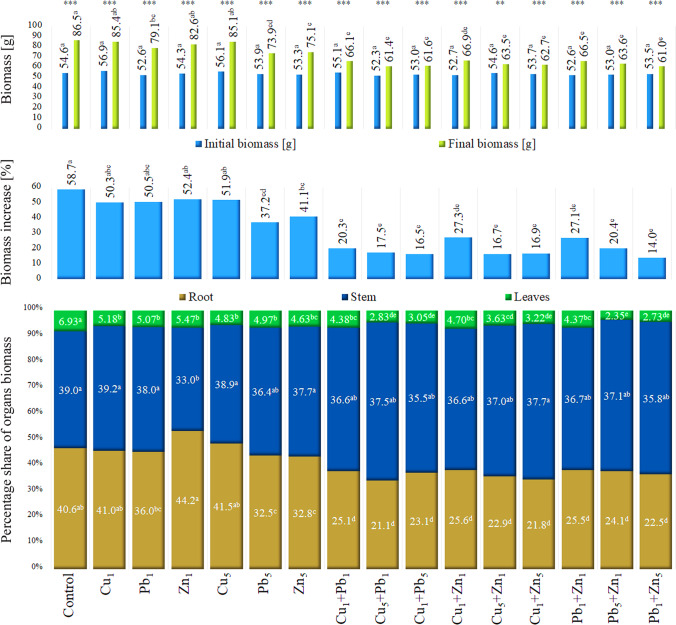
Characteristics of biomass [g] of *Acer platanoides* L. seedlings

One-year-old seedlings of *A. platanoides* L. were collected in March 2017 from the forest nursery of the Pniewy Forest Division (52^o^ 29′ 04″ N; 16 ^o^ 15′ 28″ E). All seedlings were grown in cylindrical white pots (15 × 15 cm, diameter × height) filled with unpolluted soil with pH_1M KCl_ = 7.2. The concentration of carbon (C) and nitrogen (N) was 0.27 and 3.20% of air dry mass, respectively, while the concentration of potassium (K) and phosphorus (P) was 6.21 and 2.65 mg 100 g^−1^ of soil. Before the experiment, the biomass of seedlings ranged between 52.3 and 56.9 g, with mean and median values of 53.9 and 53.6 g, respectively (Fig. 1).

The mean height of seedlings was 88 cm with extreme values of 79 and 90 cm. The seedlings used in the experiment were selected from over a thousand specimens to obtain a homogenous group of plants with a shoot diameter of 0.9–1.0 cm and a similar root system size. This criterion allowed for the unification of plants to show differences in their responses depending on the experimental systems in which they grew.

### Experiment design

Seedlings were carefully washed with tap water to remove soil particles and then placed in white cylindrical pots (18 × 19 cm, diameter × height) one plant per pot. Plants were stabilized in pots using 1.3 kg of ultrapure quartz sand (SiO_2_ content = 97%, pH = 7.2, particle size 1–3 mm). A modified Knop solution (Barabasz et al. 2010) was composed of the following chemical compounds: 0.75 mol L^−1^ of Ca(NO_3_)_2_, 0.25 mol L^−1^ of KH_2_PO_4_, 0.375 mol L^−1^ of KNO_3_, 0.312 mol L^−1^ of MgSO_4_, and also: 0.025 μmol L^−1^ of CoCl_2_, 0.025 μmol L^−1^ of CuSO_4_, 6.25 μmol L^−1^ of H_3_BO_3_, 1.25 μmol L^−1^ of KI, 0.5 μmol L^−1^ of MnCl_2_, 10 μmol L^−1^ of NaFeEDTA, 0.125 μmol L^−1^ of Na_2_MoO_4_, and 0.5 μmol L^−1^ of ZnSO_4_. This solution was used as a basic reference (control) medium with pH = 5.35 and electrical conductivity EC = 1.31 mS cm^−1^, added to each pot with the addition of 1 or 5 mmol L^−1^ of lead (II) nitrate Pb(NO_3_)_2_ and/or copper (II) nitrate trihydrate Cu(NO_3_)_2_ × 3H_2_O and/or zinc nitrate hexahydrate (Zn(NO_3_)_2_ × 6H_2_O) supplied by Sigma-Aldrich (St. Louis, MO, USA). Particular solutions contained (i) a single metal in concentrations: 1 mmol L^−1^ (Cu_1_, Pb_1_, and Zn_1_) or 5 mmol L^−1^ (Cu_5_, Pb_5_, and Zn_5_), (ii) mixed metals with 1 or 5 mmol L^−1^ of first metal (Cu, Pb or Zn) with 1 or 5 mmol L^−1^ of the second metal (Cu, Pb or Zn) addition (Cu_1_ + Pb_1_, Cu_5_ + Pb_1_, Cu_1_ + Pb_5_, Cu_1_ + Zn_1_, Cu_5_ + Zn_1_, Cu_1_ + Zn_5_, Pb_1_ + Zn_1_, Pb_5_ + Zn_1_, and Pb_1_ + Zn_5_). The pH and EC values in the individual systems were measured at the beginning of experiment only and were in ranges 5.02–5.29 for pH and 1.38–1.69 mS cm^−1^. The control system was composed of plants growing in quartz sand and modified Knop solution without metal addition. Seedlings were cultivated in sixteen experimental systems with three replications each. The experiment was conducted for 90 days in a ventilated greenhouse (mean concentration of CO_2_ = 459.2 mg L^−1^, ranging from 293 to 687 mg L^−1^, mean temperature = 22.8 °C and air relative humidity = 50.1%, with the ranges of 10.9–38.5 °C and 25.4–78.5%, respectively). All measurements were recorded automatically every hour.

### Biomass investigations

The biomass of plants was measured by weighing just before and after the experiment after drying with paper towels. Differences between the biomass of plants after and before the experiment allowed us to calculate biomass increase during the experiment. After the experiment each plant was divided into root system, stem and leaves and weighed to determine their biomass and percentage share of these parts in whole plant biomass. The root systems and leaves of plants growing in individual experimental systems were photographed and then further processed.

### Determination of Cu, Ca, K, Mg, Na, Pb and Zn in plant tissue

Samples of plant materials (roots, stems and leaves) were dried at 105 ± 5 ºC for 96 h and then ground in a Cutting Boll Mill PM 200 (Retsch, Germany). The microwave sample preparation system Mars 5 (CEM, Matthews, USA) was used for sample mineralisation. 0.500 ± 0.001 g of a dry sample was digested using 7 mL of concentrated nitric acid (Suprapure, Merck, Germany) in a closed Teflon container (180 °C, 20 min heating time, 20 min hold time, 20 min cooling). Samples were diluted with water obtained from Milli-Q system (Millipore, Germany) to a final volume of 10.0 mL and then filtered. Samples were analysed in triplicate.

The ICP-OES spectrometer Agilent 5110 (Agilent, USA) was used for metal determination. The wavelengths were 422.673 nm for Ca (radial view, atomic line), 327.395 nm for Cu (axial view, atomic line), 766.491 nm for K (radial view, atomic line), 285.213 nm for Mg (axial view, atomic line), 589.592 nm for Na (radial view, atomic line), 220.353 nm for Pb (axial view, ionic line) and Zn 213.857 nm (axial view, atomic line). The instrumental conditions used were as follows: nebuliser gas flow 0.7 L min^−1^, auxiliary gas flow 1.0 L min^−1^, plasma gas flow 12.0 L min^−1^, Radio Frequency (RF) power 1.2 kW, viewing height for radial plasma observation 8 mm, signal measurement time 5 s, 3 replicates. For calibration, commercial ICP standards in nitric acid matrix (Romil, UK) have been used. Based on physical principles on emission processes (calibration curve curvature caused by reabsorption processes) and follow ISO17025, we do not use the linearity as metrological parameter, the upper range of calibration was respectively (the radial plasma observation has been used to increase the calibration range): 1000 mg L^−1^ for Ca, 20 mg L^−1^ for Cu, 1000 mg L^−1^ for K, 500 mg L^−1^ for Mg, 1000 mg L^−1^ for Na, 20 mg L^−1^ for Pb, 20 mg L^−1^ for Zn. The detection limits (DL) were determined: 0.21 mg L^−1^ for Ca, 0.00047 mg L^−1^ for Cu, 0.34 mg L^−1^ for K, 0.00051 mg L^−1^ for Mg, 0.16 mg L^−1^ for Na, 0.0021 mg L^−1^ for Pb, 0.00062 mg L^−1^ for Zn. The method detection limits (MDL) were determined calculated based on sample preparation, respectively: 4.2 mg kg^−1^ for Ca; 0.01 mg kg^−1^ for Cu; 6.8 mg kg^−1^ for K; 0.01 mg kg^−1^ for Mg; 3.2 mg kg^−1^ for Na; 0.04 mg kg^−1^ for Pb, 0.01 mg kg^−1^ for Zn. The uncertainty for the entire analytical procedure including sample preparation was 6.4% for Ca, 9.6% for Cu, 5.3% for K, 4.5% for Mg, 3.4% for Na, 4.2% for Pb and 3.5% for Zn, respectively. Traceability was checked using certified reference material (NCSDC 73,349) with satisfactory recovery (80–120%). The results have been given for dry mass of sample.

### Analysis of total phenolic content

Total phenolic (TP) content was determined according to the Folin–Ciocalteu assay (Singleton and Rossi, 1965) with some modifications. The extraction of phenolics from leaves was carried out for homogenised samples using 80% methanol (v/v). The mixture was sonicated for 30 min, shaken for 5 h with an orbital shaker and centrifuged for 15 min at room temperature. The supernatants were evaporated to dryness. The dried residue was stored at 24 °C. The extracts were then dissolved in 1 mL of 80% methanol, and 100 µL of the extract was mixed with 100 µL of Folin–Ciocalteu reagent (diluted with H_2_O; 1:1, v/v), after 3 min 3 mL of 20% Na_2_CO_3_ were added. The mixture was incubated in darkness at room temperature for 30 min. The absorbance was measured at λ = 765 nm with a Cary 300 Bio UV–VIS scanning spectrophotometer (Varian, Palo Alto, CA). The results were expressed as mg of gallic acid equivalent (GAE) per g of fresh tissue weight (FW).

### Statistical analysis

Statistical analyses were performed using the agricolae package (R, Bell Laboratories). One-dimensional analysis of variance (ANOVA), and finally the multiple comparisons Tukey’s HSD test were used to confirm the existence of uniform (α = 0.05) groups of objects (plants growing under particular experimental systems) with respect to their biomass, element concentration in plant parts or whole plant biomass and total phenolics separately. To show differences between objects as regards the content of all determining elements, Principal Component Analysis (PCA) was performed (Falniowski 2003). In this analysis, mathematical models are formulated in the form of linear equations. This is an orthogonal transformation of the observed variables into a new set of uncorrelated variables, that is, components. Due to the fact that the variables Ca, K, Mg, Na, Cu, Pb, Zn are expressed in units of different order, the principal components analysis was performed using the correlation matrix. Heatmaps extended the graphical presentation to show similarities/differences between objects (Galili 2015). To show the diversity of plants cultivated in individual experimental systems due to the content of major elements, the rank-sum was performed separately for particular plant parts and the whole plant biomass.

## Results

### Biomass of A. platanoides seedlings

The mean biomass of seedlings before and after the experiment is shown in Fig. 1. There were no significant differences between systems in plant biomass at the experiment starting point. After the experiment, the mean biomass of seedlings was 71.3 g (median 66.7 g).

The highest total biomass was observed for control seedlings (86.5 g); lower values were noted for plants growing under the Cu_1_, Cu_5_ and Zn_1_ systems (85.4; 85.1 and 82.6 g, respectively). A significantly lower biomass of *A. platanoides* seedlings was observed for Pb_1_, Pb_5_ and Zn_1_ systems (79.1, 73.9 and 75.0 g, respectively), and the lowest and most similar biomass was determined for the remaining treatments.

**Fig. 2:**
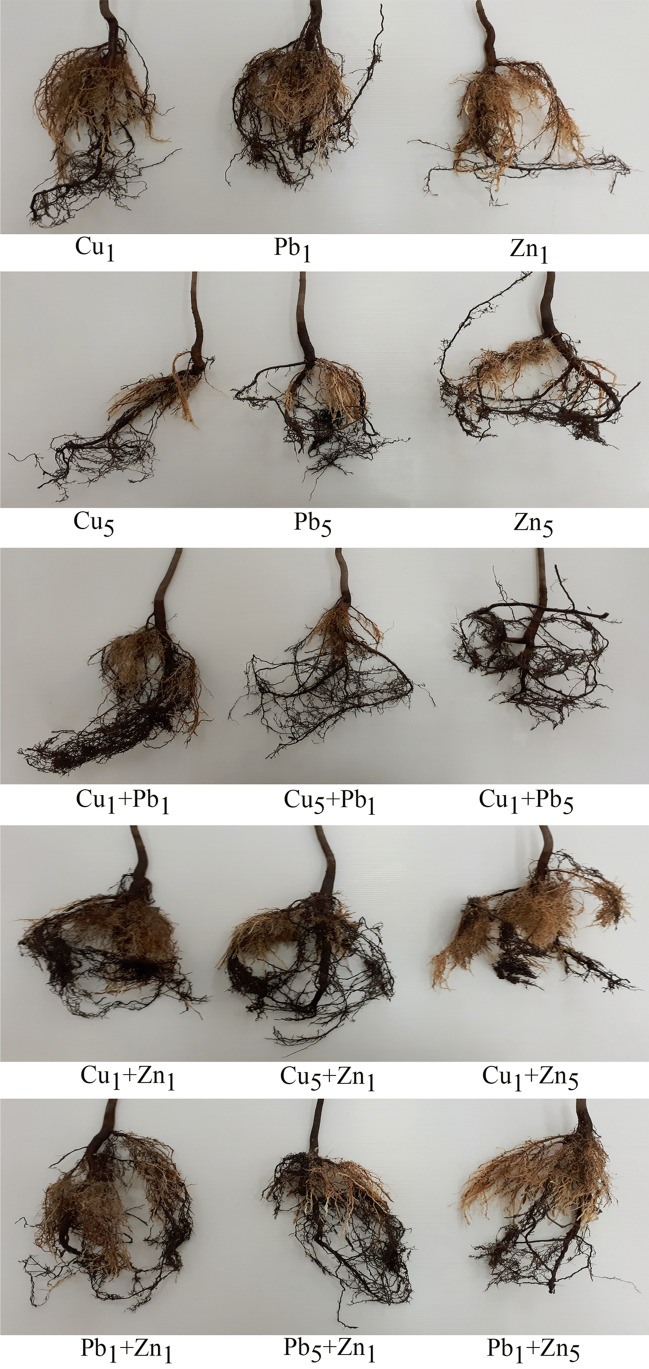
Characteristics of *Acer platanoides* L. root exposed to particular experimental systems

**Fig. 3:**
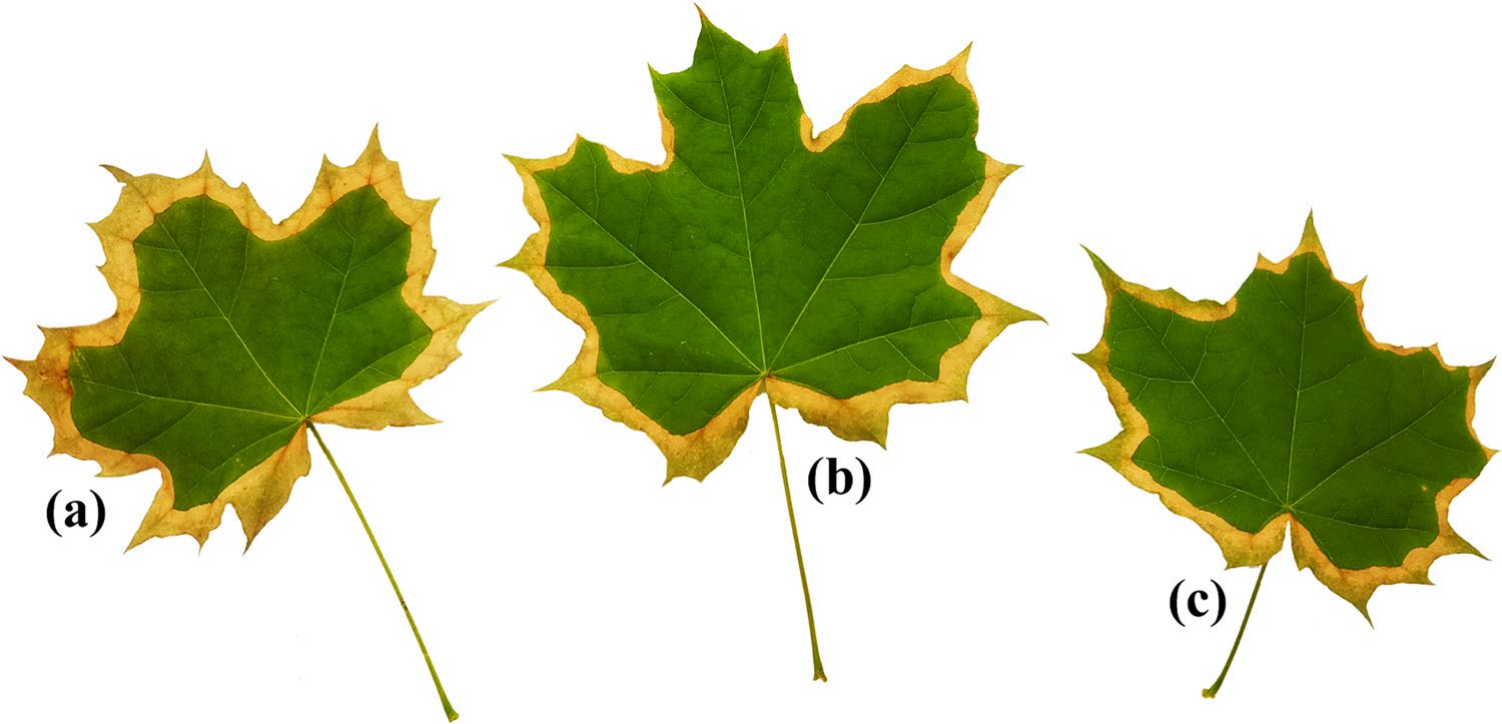
Characteristics of *Acer platanoides* L. leaves after exposition to (**a**) Pb_5_ + Zn_1_, (**b**) Pb_1_ + Zn_1_ and (**c**) Pb_1_ + Zn_5_

The mean biomass of roots, stem and leaves measured after the experiment was 30.0, 37.0, and 4.27 g, respectively. Biomass increases during the experiment of the control, Cu_1_, Pb_1_, Zn_1_ and Cu_5_-treated plants were similar (58.7, 50.3, 50.5, 52.5 and 51.9%, respectively), lower values were noted for plants exposed to Pb_5_ and Zn_5_ (37.2 and 41.4%, respectively) and the lowest for the remaining variants. In general, along with the increase of metal concentration in the Knop medium, a decrease of roots and leaves was observed with a simultaneous increase of stem biomass (Figs. 2 and 3).

The leaves of *A. platanoides* cultivated in the Pb_5_ + Zn_1_, Pb_1_ + Zn_1_ and Pb_1_ + Zn_5_ systems were characterised by specific browning of leaf edges (Fig. 3a–c). The leaves were also ~ 20% smaller than for other treatments.

### Distribution of Cu, Pb and Zn in A. platanoides parts and whole biomass

#### Metal concentration in roots

Mean Cu concentration in roots of Cu_1_ and Cu_5_-treated plants was 25.2 and 51.4 mg kg^−1^, respectively (Table 1). For plants exposed to Cu_1_, the addition of Pb decreased the Cu concentration (18.3 and 14.7 mg kg^−1^, respectively for Cu_1_ + Pb_1_ and Cu_1_ + Pb_5_). In contrast, the addition of Zn did not cause any significant changes in Cu concentration in roots. In the case of plants exposed to Cu_5_, the addition of Pb significantly limited Cu accumulation (26.7 mg kg^−1^), while the addition of Zn significantly stimulated Cu uptake (62.1 mg kg^−1^). The roots of *Acer platanoides* plants exposed to Pb_1_ and Pb_5_ contained 17.9 and 47.1 mg kg^−1^ of Pb, respectively. The addition of both Cu and Zn reduced Pb uptake, regardless of Pb concentration in the Knop solution. The roots of plants growing in the Zn_1_ and Zn_5_ systems were characterised by a mean concentration of Zn of ~ 481 and 1380 mg kg^−1^, respectively. In the case of Zn_1_, the addition of Cu caused the intensification of Zn uptake to 1190 mg kg^−1^ in the Cu_1_ + Zn_1_ system. Exposure to higher Cu concentration (Cu_5_ + Zn_1_) also significantly increased Zn uptake, but to a lesser extent (828 mg kg^−1^). The growth of plants under Zn_5_ with the addition of Cu_1_ caused stimulation of Zn uptake, while the addition of Pb_1_ did not change the Zn content in roots.

**Table 1:** Content of Cu, Pb and Zn in *Acer platanoides* organs growing under experimental systems

Experimental system	Root			Stem			Leaves		
	Cu	Pb	Zn	Cu	Pb	Zn	Cu	Pb	Zn
Control	8.70^f^	5.23^e^	10.8^e^	6.46^d^	3.33^e^	9.57^ g^	6.99^d^	3.59^c^	9.15^d^
Cu_1_	25.2^ cd^	5.52^de^	10.1^e^	18.6^bc^	3.41^e^	10.7^ g^	20.2^bc^	3.58^c^	9.24^d^
Pb_1_	8.10^f^	17.9^c^	11.3^e^	6.16^d^	5.59^de^	9.00^ g^	6.81^d^	9.52^bc^	9.08^d^
Zn_1_	8.03^f^	5.30^de^	481^d^	6.70^d^	3.71^e^	658^bc^	6.94^d^	3.63^c^	475^bc^
Cu_5_	51.4^b^	5.63^de^	10.8^e^	71.7^a^	3.90^e^	12.8^ g^	52.7^a^	3.86^c^	10.2^d^
Pb_5_	8.28^f^	47.1^a^	10.3^e^	6.03^d^	17.8^a^	12.4^ g^	6.91^d^	23.6^a^	9.89^d^
Zn_5_	8.18^f^	5.52^de^	1380^b^	6.63^d^	3.66^e^	1300^a^	7.02^d^	3.73^c^	1070^a^
Cu_1_ + Pb_1_	18.3^de^	10.2^d^	10.0^e^	16.2^c^	8.26^c^	10.3^ g^	15.8^bcd^	7.67^bc^	9.30^d^
Cu_5_ + Pb_1_	26.7^c^	8.35^de^	12.0^e^	18.3^bc^	4.80^e^	8.56^ g^	20.1^bc^	5.49^bc^	9.43^d^
Cu_1_ + Pb_5_	14.7^ef^	23.0^b^	11.1^e^	14.0^ cd^	13.2^b^	9.52^ g^	15.0^ cd^	14.5^ab^	9.56^d^
Cu_1_ + Zn_1_	24.7^ cd^	5.35^de^	1190^b^	25.3^b^	4.27^e^	806^b^	24.9^b^	3.92^c^	813^ab^
Cu_5_ + Zn_1_	62.1^a^	6.42^de^	828^c^	65.9^a^	3.96^e^	430^d^	57.0^a^	4.21^c^	572^bc^
Cu_1_ + Zn_5_	28.1^c^	6.01^de^	1790^a^	18.1^bc^	3.76^e^	782^b^	23.0^bc^	4.11^c^	1050^a^
Pb_1_ + Zn_1_	7.94^f^	8.39^de^	570^d^	6.58^d^	7.78^ cd^	506^ cd^	6.88^d^	6.69^bc^	441^bc^
Pb_5_ + Zn_1_	6.70^f^	17.1^c^	453^d^	6.87^d^	12.9^b^	373^d^	6.67^d^	11.5^bc^	335^ cd^
Pb_1_ + Zn_5_	8.30^f^	7.08^de^	1350^b^	7.06^d^	3.50^e^	705^b^	7.23^d^	4.66^c^	808^ab^

*n* = 3; identical superscripts (a, b, c) denote non-significant differences between means in columns according to the post-hoc Tukey’s HSD test

**Fig. 4:**
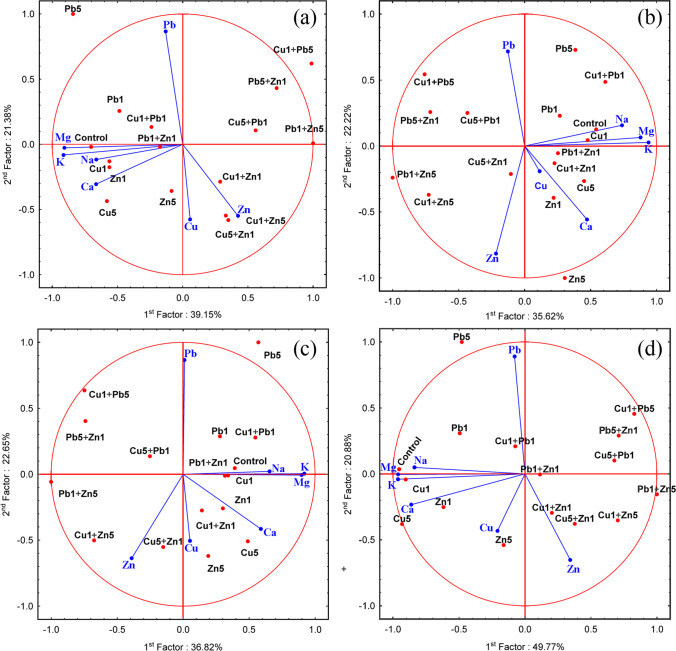
Principal Component Analysis concerning the content of determined elements in roots (**a**), stem (**b**), leaves (**c**) and whole plants (**d**) of *Acer platanoides* L

The PCA analysis revealed that in the case of roots, 60.53% (39.15 + 21.38) of total variability was explained, which indicates similarities/differences between the studied objects (plants cultivated in particular experimental systems) (Fig. 4a). Strong positive correlation between content of Mg, K, Na, and Ca was observed. Additionaly, there was no correlation between the content of Pb, Cu and Zn in relation to mentioned major elements. A positive correlation between content of major elements and control, Cu_1_, Pb_1_, Zn_1_, Cu_5_, Zn_5_, Cu_1_ + Pb_1_, and Pb_1_ + Zn_1_ was observed, while a negative for Cu_1_ + Pb_5_, Cu_1_ + Zn_1_, Cu_1_ + Zn_5_, Cu_5_ + Zn1, Pb1 + Zn_5_, Cu_5_ + Pb_1_, and Pb_5_ + Zn_1_. Three similar groups of objects are clearly visible and characterised by the highest concentrations of Cu (Cu_5_ and Cu_5_ + Zn_1_), Pb (Pb_5_ and Cu_1_ + Pb_5_) and Zn (Zn_5_, Cu_1_ + Zn_1_, Cu_1_ + Zn_5_ and also Pb_1_ + Zn_5_).

#### Metal concentration in stem

The concentration of Cu in plants exposed to Cu_1_ and Cu_5_ reached 18.6 and 71.7 mg kg^−1^, respectively (Table 1). The addition of Pb_1_, Pb_5_, Zn_1_ or Zn_5_ to Knop solution enriched with Cu_1_ did not cause any significant changes in Cu content in *A. platanoides* stems. On the other hand, the addition of Pb_1_ significantly decreased Cu content in stems, and no changes in Cu uptake were noted for Zn_1_ addition. The stems of *A. platanoides* cultivated in the Pb_1,_ or Pb_5_ systems were characterised by a Pb content of 5.59 and 17.8 mg kg^−1^, respectively. Significant stimulation of Pb uptake by stems was observed for the Cu_1_ + Pb_1_ system (8.26 mg kg^−1^), while no significant changes were noted for Cu_5_ + Pb_1_, Pb_1_ + Zn_1_ and Pb_1_ + Zn_5_-treated plants. The stems of *A. platanoides* growing under Pb_5_ with the addition of Cu_1_ or Zn_1_ were characterised by significantly lower Pb uptake (13.2 and 12.9 mg kg^−1^, respectively) compared to Pb_1_. The content of Zn in the stems of plants exposed to Zn_1_ and Zn_5_ reached 658 and 1300 mg kg^−1^, respectively (Table 1). Higher concentrations of Cu or Pb (Cu_5_ + Zn_1_, Pb_5_ + Zn_1_) significantly limited Zn accumulation in stems (430 and 373 mg kg^−1^, respectively). The addition of Cu_1_ or Pb_1_ together with Zn_5_ was the cause of a significant decrease of Zn concentration in *A. platanoides* stems (782 and 705 mg kg^−1^, respectively).

A PCA analysis for stems explained 57.84% (35.62 + 22.22) of the total variability (Fig. 4b).

Strong positive correlation between content of Mg, K, and Na was observed. Additionaly, there was no correlation between the content of Pb, Cu and Zn in relation to mentioned major elements. A positive correlation between content of major elements and control, Cu_1_, Pb_1_, Zn_1_, Cu_5_, Pb_5_, Zn_5_, Cu_1_ + Pb_1_, Cu_1_ + Zn_1_ and Pb_1_ + Zn_1_ was observed, while a negative for rest experimental systems. Experimental systems characterised by the highest content of Cu (Cu_5_ and Cu_5_ + Zn_1_) and Pb (Pb_5_ and Cu_1_ + Pb_5_) were placed close to each other, while experimental systems with the highest concentration of Zn (Zn_5_, Cu_1_ + Zn_1_, Cu_1_ + Zn_5_ and also Pb_1_ + Zn_5_) were dispersed.

#### Metal concentration in leaves

The leaves of *A. platanoides* plants cultivated in the Cu_1_ and Cu_5_ systems were characterised by mean concentrations of Zn of 20.2 and 52.7 mg kg^−1^, respectively (Table 1). The addition of Pb_1_, Pb_5_, Zn_1_ or Zn_5_ did not cause any significant changes in Cu content in the leaves of plants growing in the Cu_1_ system. The content of Cu in the leaves of control and Cu_5_ + Zn_1_ plants was similar (52.7 and 57.0 mg kg^−1^, respectively) and significantly higher than in the leaves of plants growing under the Cu_5_ + Pb_1_ system (20.1 mg kg^−1^). The content of Pb in the leaves of plants growing under the Cu_1_ + Pb_1_, Cu_5_ + Pb_1_, Pb_1_ + Zn_1_ or Pb_1_ + Zn_5_ systems (7.67, 5.49, 6.69 or 4.66 mg kg^−1^, respectively) was similar to its content in the leaves of plants under the Pb_1_ system (9.52 mg kg^−1^). The content of Pb in the leaves of *A. platanoides* in the Pb_5_ system reached 23.6 mg kg^−1^, while significantly lower contents were recorded for Cu_1_ + Pb_5_ or Pb_5_ + Zn_1_-treated plants (14.5 or 11.5 mg kg^−1^, respectively). The mean concentration of Zn in leaves in the Cu_1_, Cu_5_ + Zn_1_, Pb_1_ + Zn_1_, and Pb_5_ + Zn_1_ system was similar (475, 572, 441, and 335 mg kg^−1^, respectively) and significantly lower than in Cu_1_ + Zn_1_ (813 mg kg^−1^). The addition of Cu_1_ or Pb_1_ to Zn_5_ did not cause any differences in Zn uptake (1050 and 808 mg kg^−1^, respectively) compared to the Zn_5_ system (1070 mg kg^−1^).

The PCA analysis explained 59.47% (35.82 + 22.65) of the total variability of metal content in leaves (Fig. 4c) and was similar to the PCA performed for stems (Fig. 4b). Strong positive correlation between content of Mg, K, Na, and Ca was observed. Additionaly, there was no correlation between the content of Pb, Cu and Zn in relation to mentioned major elements. A positive correlation between content of major elements and control, Cu_1_, Pb_1_, Zn_1_, Cu_5_, Pb_5_, Zn_5_, Cu_1_ + Pb_1_, Cu_1_ + Zn_1_, and Pb_1_ + Zn_1_ was observed, while a negative for the rest of experimental systems. Experimental systems characterised by the highest concentration of Cu or Pb were placed close to each other. In contrast, a clear dispersion was visible for systems with the highest Zn concentration.

### Metal content in whole plant biomass

**Fig. 5:**
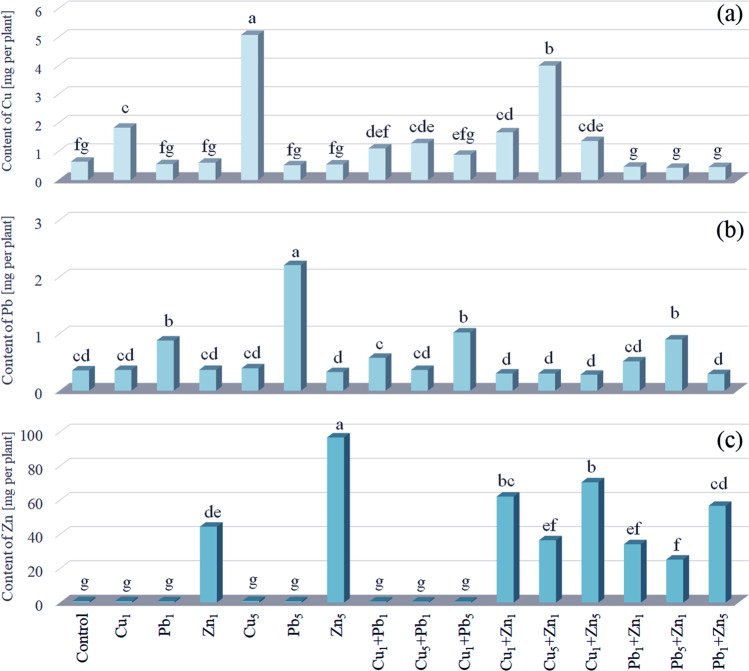
Content of Cu, Pb and Zn [mg per plant] in whole biomass of *Acer platanoides* L. seedlings

The content of Cu in the whole biomass of plants exposed to Cu_1_ reached 1.84 mg per plant, while in the Cu_1_ + Pb_1_, Cu_1_ + Pb_5_ systems, its content was significantly lower (1.11 and 0.887 mg per plant, respectively). The addition of Zn both at 1 and 5 mmol L^−1^ concentrations did not cause any significant change of Cu content in the whole plant biomass (Fig. 5).

The growth of *A. platanoides* under Cu_5_ was characterised by the effective accumulation of the metal (5.09 mg per plant). In comparison, the addition of Pb led to a significantly lower accumulation of Cu (1.29 mg per plant). The exposure of plants to Cu_5_ + Zn_1_ did not cause any significant change in Cu accumulation (4.01 mg per plant).

The content of Pb in the whole plant biomass in the Pb_1_ or Pb_5_ systems reached 0.882 and 2.21 mg per plant, respectively. The addition of Cu or Zn at 1 or 5 mmol L^−1^ concentrations resulted in a significant decrease of Pb content in the whole biomass of *A. platanoides* (Fig. 5). Cu addition in the Cu_1_ + Zn_1_ system stimulated Zn accumulation, while Pb addition in the Pb_5_ + Zn_1_ system limited Zn uptake to 62.1 and 25.2 mg per plant, respectively. Plants cultivated in the Cu_5_ + Zn_1_ and Pb_1_ + Zn_1_ systems were characterised by a similar content of Zn (36.5 and 34.2 mg per plant, respectively) as for Zn_1_ (44.5 mg kg^−1^). The addition of Cu_1_ or Pb_1_ to Zn_5_ caused a significantly lower accumulation of Zn (70.5 and 56.7 mg per plant, respectively) compared to Zn_5_ (96.7 mg kg^−1^).

PCA for metal accumulation in the whole plant biomass explained 70.65% (49.77 + 20.88) of the total variability (Fig. 4d). Strong positive correlation between content of Mg, K, Na, and Ca was observed. Additionaly, there was no correlation between the content of Pb, Cu and Zn in relation to mentioned major elements. A positive correlation between content of major elements and control, Cu_1_, Pb_1_, Zn_1_, Cu_5_, Pb_5_, and Zn_5_ was observed, while a negative for the rest experimental systems. The value highlighted the similarities between the Cu content in plants under Cu_5_ and Cu_5_ + Zn_1_, as well as in plants growing under Pb_5_ with a significantly lower content of this metal in plants under Cu_1_ + Pb_5_. In the case of plants characterised by the highest content of Zn in the whole biomass, such relations could not be found despite the proximity of individual objects (Zn_5_, Cu_1_ + Zn_1_, Cu_1_ + Zn_5_ and also Pb_1_ + Zn_5_), which probably resulted from the loss of information (29.35%) during the transition from the n- to the two-dimensional system.

### Distribution of major elements in Acer platanoides parts and whole biomass

Among the examined major elements, the highest diversity of changes was found for the content of Ca and Na in *A. platanoides* parts (Table 2). In contrast, the differences between the content of K and Mg were slight between most plants cultivated in the individual experimental systems. The highest Ca content in roots was determined for the Zn_1_ and Zn_5_ systems(24,800 and 23,800 mg kg^−1^, respectively). Plants cultivated in the Zn_5_ system were also characterised by the highest concentration of Ca in stems and leaves (23,900 and 22,100 mg kg^−1^, respectively). It is worth emphasizing that the presence of two metals in the cultivation medium caused a decrease in Ca content in *A. platanoides* parts.

**Table 2:** Content of major elements [mg kg^−1^] in *Acer platanoides* organs growing under particular experimental systems

Experimental system	Root				Stem				Leaves			
	Ca	K	Mg	Na	Ca	K	Mg	Na	Ca	K	Mg	Na
Control	20700^bcd^	17900^a^	5620^abc^	164^abc^	17300^c−f^	14400^ab^	4520^ab^	137^ab^	18200^b−e^	13700^abc^	4600^abc^	129^ab^
Cu_1_	18700^def^	17200^ab^	5440^a−d^	182^a^	18000^cd^	13900^ab^	4400^abc^	134^abc^	16900^c−g^	13600^abc^	4440^abc^	142^a^
Pb_1_	23300^ab^	15300^a−e^	5460^a−d^	128^cde^	16600^d−g^	12900^ab^	4520^abc^	118^b−e^	18800^a−d^	13500^abc^	4450^abc^	113^a−d^
Zn_1_	24800^a^	16800^abc^	5900^abc^	102^d−g^	19000^bc^	14300^ab^	4540^ab^	84^fgh^	20000^abc^	14800^ab^	4760^abc^	85^d−g^
Cu_5_	22700^abc^	15300^a−e^	6280^ab^	117^def^	20400^b^	12000^abc^	5260^a^	80^gh^	21200^ab^	12400^bcd^	5240^a^	90^d−g^
Pb_5_	18600^def^	15800^a−d^	6620^a^	161^abc^	17600^cde^	12700^ab^	5170^a^	111^b−f^	17200^c−f^	13100^abc^	5440^a^	126^abc^
Zn_5_	23800^a^	14500^a−f^	5410^a−d^	103^d−g^	23900^a^	12900^ab^	4550^ab^	100^d−g^	22100^a^	13500^abc^	4630^abc^	93^c−g^
Cu_1_ + Pb_1_	17200^e−h^	16400^abc^	5290^a−d^	139^bcd^	14800^ghi^	15600^a^	4530^ab^	153^a^	15500^d−g^	16200^a^	4710^abc^	140^a^
Cu_5_ + Pb_1_	17800^d−g^	10200^fg^	4980^a−d^	67^gh^	13100^i^	11100^bcd^	4230^abc^	57^h^	15200^efg^	10900^cde^	4700^abc^	58^g^
Cu_1_ + Pb_5_	16700^f−h^	9040^g^	3930^d^	57^h^	14500^hi^	8760^cd^	3730^bc^	63^h^	14300^fg^	8930^de^	3730^bc^	59^fg^
Cu_1_ + Zn_1_	15300^gh^	12800^b−g^	4930^bcd^	170^ab^	13500^i^	13800^ab^	4600^ab^	125^a−d^	14100^fg^	13200^abc^	4790^ab^	141^a^
Cu_5_ + Zn_1_	18600^def^	12400^c−g^	4880^bcd^	118^def^	15600^fgh^	10900^bcd^	4190^abc^	102^c−g^	16300^d−g^	10300^cde^	4480^abc^	111^a−e^
Cu_1_ + Zn_5_	20000^cde^	11300^d−g^	5190^a−d^	134^b−e^	15900^e−h^	8200^cd^	3580^bc^	81^fgh^	17100^c−f^	8240^e^	3830^bc^	94^c−f^
Pb_1_ + Zn_1_	20700^bcd^	17200^ab^	5030^a−d^	107^def^	17900^cd^	13800^ab^	4850^ab^	88^e−h^	18100^b−e^	14800^ab^	4790^ab^	102^b−e^
Pb_5_ + Zn_1_	15500^gh^	10600^fg^	4360^cd^	98^efg^	16500^d−g^	8420^cd^	3640^bc^	77^gh^	14900^efg^	8580^e^	3620^bc^	82^d−g^
Pb_1_ + Zn_5_	14300^ h^	11100^efg^	4320^cd^	78^fgh^	14200^hi^	7820^d^	3130^c^	76^gh^	13400^g^	8430^e^	3450^c^	77^efg^

*n* = 3; identical superscripts (a, b, c) denote non-significant differences between means in columns according to the post-hoc Tukey’s HSD test

The highest content of Na was observed in the roots of plants cultivated in the Cu_1_ and Cu_1_ + Zn_1_ systems (182 and 170 mg kg^−1^, respectively) and stems of plants in the Cu_1_ + Pb_1_ system (153 mg kg^−1^). In leaves, the highest content of Na was determined for all three mentioned experimental systems (Cu_1_, Cu_1_ + Pb_1_, and Cu_1_ + Zn_1_), reaching 142, 140 and 141 mg kg^−1^, respectively. Generally, K content was lower in plant parts co-treated with the remaining metals.

The analysis of the content of major elements in whole plant biomass revealed the highest Ca content in *A. platanoides* cultivated in the Zn_1_, Cu_5_ and Zn_5_ systems (1800, 1830 and 1770 mg kg^−1^, respectively), while Na content was the highest in control seedlings and the Cu_1_ system (12.6, and 13.2 mg kg^−1^, respectively). Overall, the total content of all four metals in the total plant biomass decreased with the appearance of the second metal in the Knop medium (Table 3).

**Table 3:** Content of major elements [mg per plant] in whole biomass of *Acer platanoides*

Experimental system	Ca	K	Mg	Na
Control	1630^b^	1350^a^	430^ab^	12.6^a^
Cu_1_	1540^b^	1290^ab^	412^abc^	13.2^a^
Pb_1_	1550^b^	1100^abc^	385^a−d^	9.6^b^
Zn_1_	1800^a^	1290^ab^	431^ab^	7.6^b−e^
Cu_5_	1830^a^	1150^abc^	485^a^	8.3^bcd^
Pb_5_	1320^c^	1030^bc^	426^abc^	9.8^b^
Zn_5_	1770^a^	1020^bc^	367^b−e^	7.5^b−e^
Cu_1_ + Pb_1_	1040^de^	1060^abc^	318^c−f^	9.7^b^
Cu_5_ + Pb_1_	908^fg^	664^ef^	278^def^	3.7^g^
Cu_1_ + Pb_5_	938^efg^	547^f^	234^f^	3.7^g^
Cu_1_ + Zn_1_	950^efg^	892^cde^	317^c−f^	9.5^bc^
Cu_5_ + Zn_1_	1050^de^	713^def^	282^def^	6.9^c−f^
Cu_1_ + Zn_5_	1090^d^	573^f^	257^ef^	6.2^d−g^
Pb_1_ + Zn_1_	1250^c^	1002^bcd^	325^b−f^	6.5^def^
Pb_5_ + Zn_1_	1020^def^	585^def^	247^f^	5.4^efg^
Pb_1_ + Zn_5_	862^g^	548^g^	217^f^	4.7^fg^

*n* = 3; identical superscripts (a, b, c) denote non-significant differences between means in columns according to the post-hoc Tukey’s HSD test

**Fig. 6:**
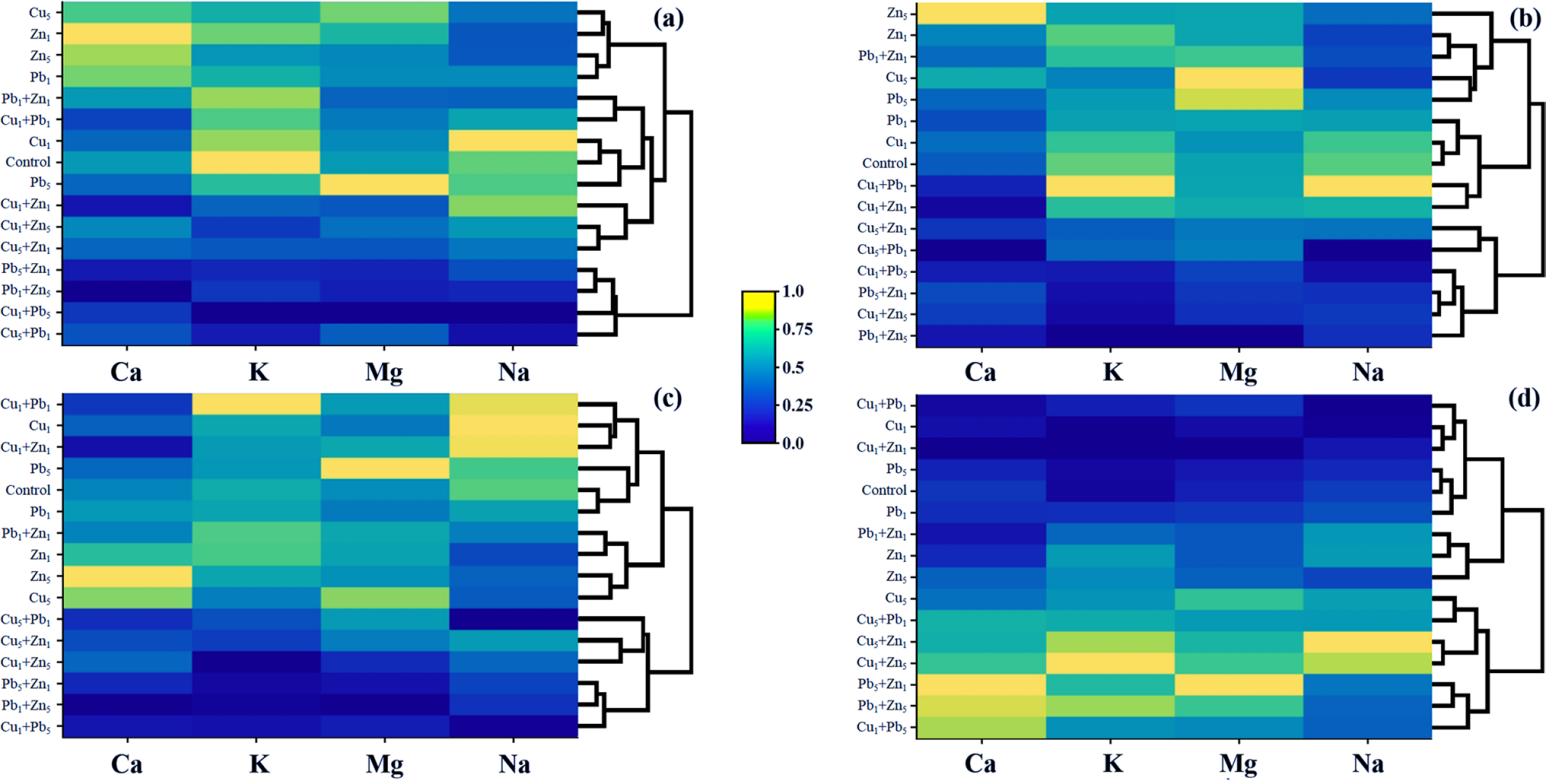
Correlation between experimental systems considering the content of major elements jointly (Heatmap) in *Acer platanoides* L. presented as a hierarchical tree plot

Heatmaps were performed to compare the content of major elements, in particular plant parts and whole plant biomass. For roots, three separate groups of objects were separated: (i) Cu_5_, Zn_1_, Zn_5_, and Pb_1_; (ii) Pb_5_ + Zn_1_, Pb_1_ + Zn_5_, Cu_1_ + Pb_5_, and Cu_5_ + Pb_1_, (iii) remaining systems (Fig. 6a). In the case of stems, the following separate groups of objects were observed: (i) Cu_5_, Pb_5_, Zn_1_, Zn_5_, and Pb_1_ + Zn_1_; (ii) Control, Cu_1_, Pb_1_, Cu_1_ + Pb_1_, Cu_1_ + Zn_1_ and (iii) remaining systems (Fig. 6b). Finally, for leaves three groups of objects were also separated: (i) Control, Cu_1_, Pb_1_, Pb_5_, Cu_1_ + Pb_1_, and Cu_1_ + Zn_1_; (ii) Cu_5_, Zn_1_, Zn_5_, and Pb_1_ + Zn_1_; and (iii) remaining systems (Fig. 6c). A heatmap prepared for the concentration of all major elements in the whole plant biomass revealed three groups of objects: (i) Zn_1_, Zn_5_, and Cu_5_; (ii) Control, Cu_1_, Pb_1_, Pb_5_, Cu_1_ + Pb_1_, and Cu_1_ + Zn_1_; and (iii) remaining systems (Fig. 6d). It is worth underlining that in the second-mentioned group, two different groups could be indicated: the first included: Control, Pb_1_ and Pb_5_, and the second contained: Cu_1_, Cu_1_ + Pb_1_ and Cu_1_ + Zn_1_, which may suggest that the content of major elements depends on the presence of a particular element.

**Fig. 7:**
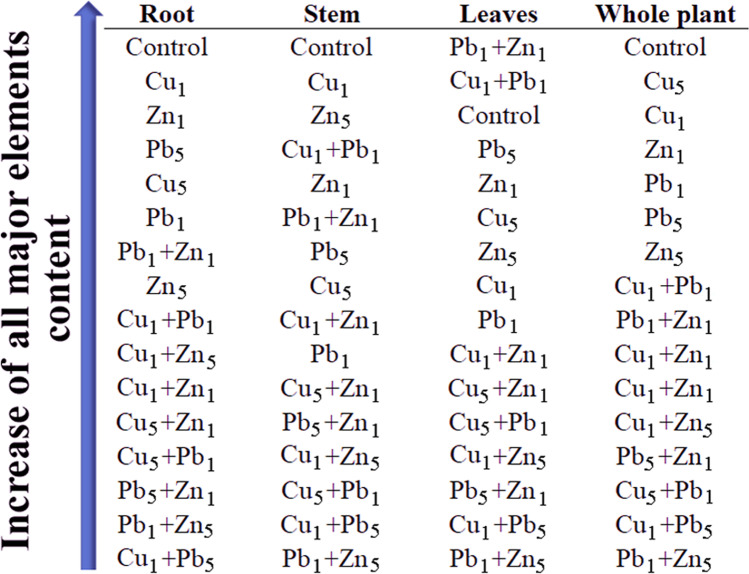
Graphical presentation of rank-sum according to increase of major elements in roots, stem, leaves and whole plants of *Acer platanoides* L

The previous observation is partially confirmed by the rank sum analysis, where the content of major elements was arranged following increasing values (Fig. 7). Considering the whole biomass, rank-sum indicated that the content of the determined major element was the lowest when the plant was exposed to two metals simultaneously. Moreover, plants growing under Cu (both Cu_1_ and Cu_5_) were characterised by a higher content of major elements than plants exposed to Pb or Zn. A rank-sum for plant parts showed a similar dependence.

### Phenolic content in A. platanoides leaves

**Fig. 8:**
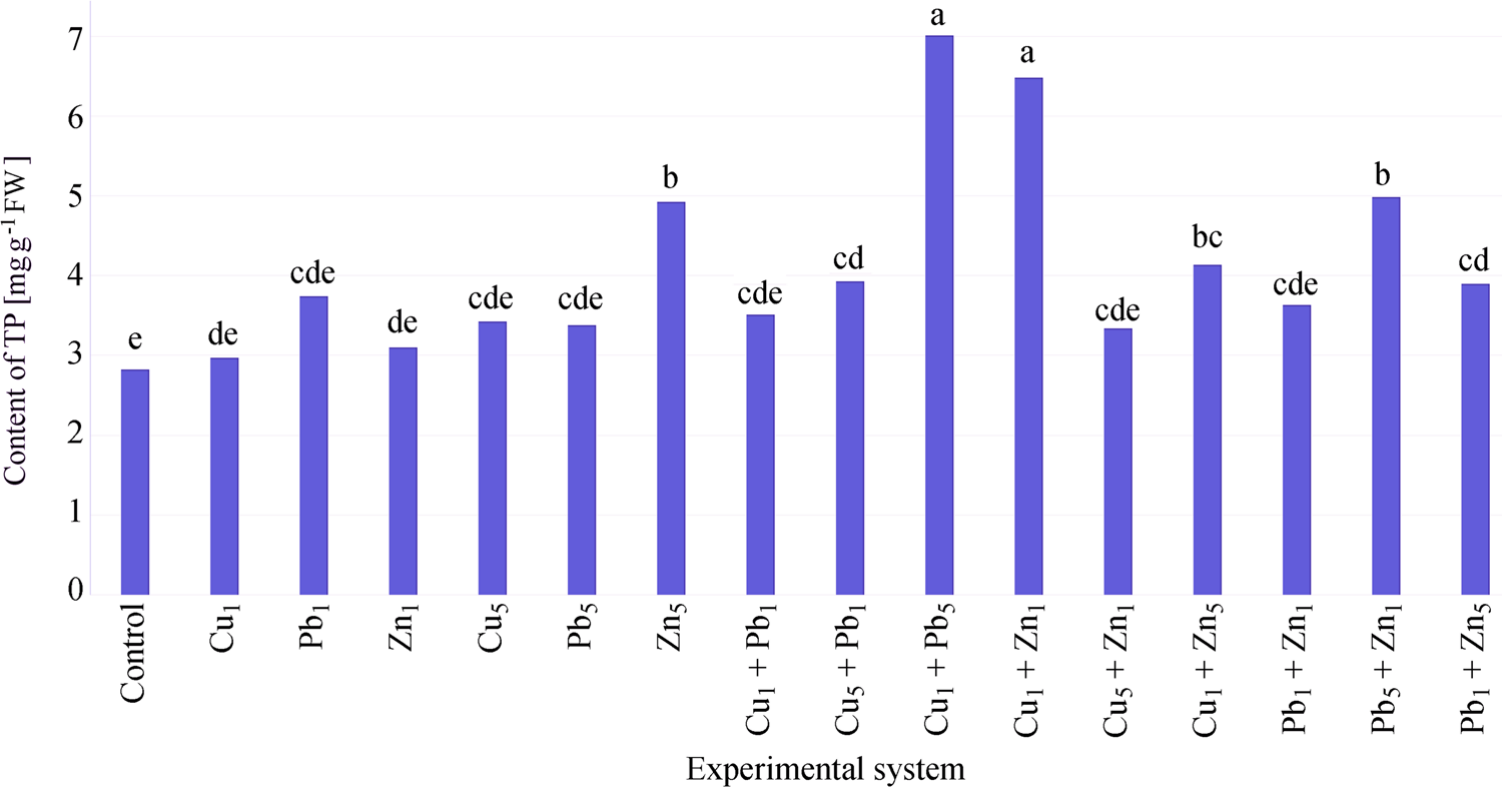
Total phenolic content in leaves of *Acer platanoides* L

Total phenolic (TP) content in leaves varied between applied cultivation systems. The lowest TP was noted in the control (2.82 mg g^−1^ FW). The addition of Cu, Pb, Zn to the substrate resulted in the elevation of TP content (Fig. 8). The highest TP was confirmed for Cu_1_ + Pb_5_ (7.01 mg g^−1^ FW) and Cu_1_ + Zn_1_ (6.48 mg g^−1^ FW), followed by Pb_5_ + Zn_1_ and Zn_5_ (4.98 and 4.92 mg g^−1^ FW, respectively). TP content did not exceed 4.20 mg g^−1^ FW in other experimental systems. The addition of 1 and 5 mmol L^−1^ of Cu, Pb and 1 mmol L^−1^ of Zn caused a slight (not significant) increase in TP.

A slight elevation of TP was also confirmed for Cu_1_ + Pb_1_, Cu_5_ + Zn_1_, Pb_1_ + Zn_1_ in comparison to the control. A significant rise of TP was confirmed for Zn_5_, Cu_1_ + Pb_5_, Cu_5_ + Pb_1_, Cu_1_ + Zn_1_, Cu_1_ + Zn_5_, Pb_5_ + Zn_1_, Pb_1_ + Zn_5_ in comparison to the control. The highest TP content was observed in the Cu_1_ + Pb_5_ and Cu_1_ + Zn_1_ experimental systems. TP content was higher in the leaves of plants simultaneously treated with two metals than for a single metal applied in equivalent concentrations for Cu_1_, Pb_5_ and Zn_1_ bi-metal combinations. TP content for Cu_1_ + Zn_5_ was significantly higher than Cu_1_ and comparable to Zn_5_. For Pb_5_ + Zn_1,_ TP was significantly higher than for Pb_5,_ and Zn_1_ added individually.

## Discussion

### Metal interactions and plant growth

According to Luo and Rimmer (1995), who analysed the interactions between Cd, Cu, Pb and Zn in spring barley, plant growth depended mainly on the amount of Zn available for the plant and the concentration of Cu. The significant role of Zn in stimulating or inhibiting the growth of barley was also pointed out by Sanders and Adams (1987) and Sanders et al. (1987), emphasizing the no less important role of the soil pH and the addition of organic matter. The straightforward but differentiated interaction between Cu and Zn has been described many times. Le et al. (2013), examining the mutual interaction of both elements, found that the presence of Zn reduced the toxicity of Cu, while the presence of Cu did not affect the toxicity of Zn. Montvydienė and Marčiulionienė (2007) reached similar conclusions. In the research presented in this paper, adding Cu to Knop solution containing Zn or Zn to Cu caused a significantly lower biomass of *A. platanoides* seedlings. This result is consistent with the observations of Luo and Rimmer (1995), who suggested that the addition of Cu to the medium increased the toxicity of Zn. Lower biomass in the case of seedling growth in experimental systems containing higher concentrations of Cu, Pb or Zn is not surprising. It is usually associated with modifications of the root system (Krzesłowska et al. 2019). The presence of more than one element can stimulate a toxic effect, inducing changes in plant ultrastructure, as clearly demonstrated by Xiao et al. (2010), who studied the damage of wheat seedlings growing under Cu and Pb.

The importance of the optimal Zn content in the plant is crucial as the metal is the main component of ribosomes and is necessary for the production of proteins in plants (Sinclair and Krämer 2012). Unfortunately, phosphorus (P) present in the nutrient solution and soil eagerly interacts with Zn, modifying its availability, which in many cases can be interpreted as interaction with Cu. In the performed experiment, the level of P was similar in all experimental systems, and only its increase reduces the accumulation of Zn by plants (Mousavi et al. 2012). The presence of two elements in the substrate in varying concentrations may inhibit plant growth in various ways, which results from their different phytotoxicity (Fargašová 2004). In addition, the interpretation of the basal relationships between bivalent ions that often use the same transporters is challenging due to the interactions of the ion network (Andresen et al. 2018). An example is the interaction of Fe and Zn, where Zn is transported via root cells using the ZIP transporter family (Walker and Connolly 2008). On the other hand, transporters binding Fe may also bind Zn. The effect may be the increased transport of both metals, which may elevate the toxicity and adversely affect plant growth (Rout and Das 2003).

### Metal interactions and uptake

Studies on the interactions between elements are complicated due to their complexity and, at the same time, it is extremely important to determine the possible impact of the presence of another element on the uptake of a specific pollutant present in soil (Adamczyk-Szabela et al. 2020; Fargašová and Beinrohr 1998; Nlemadim et al. 2019). This problem is crucial because we can only talk about specific metal–metal interactions by examining plants under controlled conditions and using possibly high and non-toxic doses of their salts to avoid the possible influence of other components present in the soil or the nutrient solution used (Fargašová et al. 2006). The use of too high concentrations of metals in the substrate does not allow the interaction to be determined owing to too high toxicity (Nicholls and Mal 2003). The presence of many other elements, such as iron (Fe) or manganese (Mn), can significantly affect the accumulation of Cu, Pb and Zn (Ghasemi-Fasaei and Ronaghi 2007). For this reason, it is possible to obtain different results, as pointed out by Luo and Rimmer (1995). Describing the interactions between Cu and Zn in spring barley, the authors indicated an evident synergism, while Kabata-Pendias and Pendias (2001) noted an antagonism between these metals. This dualism in the interpretation of research results is an effect of the influence of other factors, such as the level of the cation-exchange capacity (CEC). According to Qiu et al. (2016), a high and medium level of CEC was the cause of synergistic relations between these elements in *Hordeum vulgare*, while a low level of CEC was characteristic for antagonistic interactions. The authors pointed to the competition of Cu and Zn ions for their binding by plant roots. Interactions play an essential role not only in stimulating or inhibiting the uptake of specific elements but can also affect the action of organic compounds present in the medium and even be responsible for the evolution of hyperaccumulation (Jhee et al. 2006). Taking this reasoning as a starting point, and the fact that using a nutrient medium or soil containing many other chemical compounds in research, it is possible to modulate the mutual relations between Cu and Zn, not only limited to those mentioned (Singh et al. 2016).

The performed experiment showed that the content of Cu in roots of *A. platanoides* exposed to Cu_1_ was similar to Cu_1_ + Zn_1_ and Cu_1_ + Zn_5_ (25.2, 24.7 and 28.1 mg kg^−1^, respectively), while it was lower when Zn ions were replaced with Pb in the medium (18.3 and 14.7 mg kg^−1^, respectively) (Table 1). At higher concentrations of Cu in the medium (Cu_5_), the uptake of Cu was restricted in plants additionally exposed to Pb ions (Cu_5_ + Pb_1_) and increased after the addition of Zn ions (Cu_5_ + Zn_1_) (51.4, 26.7 and 62.1 mg kg^−1^, respectively). These results clearly indicate the antagonism between Cu and Pb and the interaction between Cu and Zn dependent on the concentration of both metals in the medium. It should be emphasized that the concentration of Zn in roots of *A. platanoides* cultivated in the Zn_1_ system was significantly lower than for plants in the Cu_1_ + Zn_1_ and Cu_5_ + Zn_1_ systems (481, 1190 and 828 mg kg^−1^, respectively) (Table 1). The visible synergistic effect of Cu decreased with an increase in its concentration in the Knop medium, which indicated that lower concentrations of Cu may perpetuate the uptake of Zn. At the same time, too high concentrations may inhibit the accumulation of this metal and possibly reduce plant biomass. Similar observations were also found for animal species, such as *Lymnaea stagnalis* in the studies of Cremazy et al. (2018), who pointed to additive effect between Cu and Zn. It is worth noting that Zn accumulation by plants cultivated in the Zn_5_ system was similar to Pb_1_ + Zn_5_-treated plants and significantly higher in the Cu_1_ + Zn_5_ system (1380, 1350 and 1790 mg kg^−1^, respectively). Therefore, it can be assumed that the dualism observed in various studies resulted from different quantitative ratios of the metals and perhaps other factors influencing their uptake (Luo and Rimmer 1995; Le et al. 2013). The results obtained in this experiment clearly indicate the antagonism between the Pb and Cu as well as Pb and Zn ions in the roots of *A. platanoides*, which is consistent with the observations of He et al. (2004) and Musielińska et al. (2016).

The mutual quantitative relations of metals present in the substrate are essential factors influencing their accumulation. They can completely change the type of interaction, as exemplified by the studies of Feng et al. (2009). The authors, examining the influence of arsenic (As) and selenium (Se) on *Pteris vittata* L., indicated that stimulation or inhibition of As uptake in Chinese brake fern was observed depending on the concentration of Se. These observations explain the differences in the concentration of metals observed in our experiment, especially in plant roots, depending on the applied experimental system. Assessment of interactions for stems, and especially leaves, is hardly possible because still little is known about the forms of elements transported to the above-ground parts and their interactions within the plant (Peralta-Videa et al. 2009). Moreover, the differences in the Cu, Pb and Zn translocations can be significant (transport of Cu and Zn with limitation of Pb mainly in the root system), which allows only an indication of a higher/lower content of these metals in a specific parts of the tree (Eapen and D'souza 2005; Musielińska et al. 2016).

### Phenolic accumulation

Phenolic compounds are recognised as molecules playing a pivotal role in stress tolerance and plant protection from oxidative stress through scavenging of free-radical and neutralisation of reactive oxygen species (ROS), inhibition of lipid oxidation, being osmoprotectants and metal chelators (Ahmad et al. 2015; Hadi et al. 2016; Isbilir and Sagiroglu 2013; Maslennikov et al. 2018). It was documented that leaves of plants growing on a polluted site contained a higher content of phenolic compounds in comparison to those growing in clean conditions (Ahmad et al. 2015; Ullah et al. 2019). An increase of TP content in leaves was observed as a response to stress conditions and contamination of different environment components (Cannac et al. 2007; Robles et al. 2003; Yang et al. 2007; Azzazy 2020).

Thus this parameter was postulated as a major biochemical indicator of stress factors (Azzazy 2020). However, the toxicity of Cu, Pb and Zn in lower concentrations did not affect the total phenolic content. The stimulation of TP content in the experimental systems by higher concentration (5 mmol L^−1^) was confirmed only for Zn. In this experimental system, the Zn content was highest. Thus, the stimulation of phenolic content was a reaction to increasing the element. No effect was confirmed for elevated Pb and Cu. The simultaneous addition of both elements at low concentration resulted in a significant elevation of phenolic compound synthesis only for Cu_1_ + Zn_1_. In this experimental system, the content of Cu and Zn in leaves was significantly higher than in the control and for Cu_1_ and Zn_1_ separately.

A significant increase of TPC was observed for plants treated with two metals simultaneously, i.e. Cu_1_ + Pb_5_ (highest TPC), Pb_5_ + Zn_1_, Pb_1_ + Zn_5_, Cu_5_ + Pb_1_. It indicated that the synthesis of phenolic compounds can be increasingly stimulated by combining two metals rather than by a single one. The results suggested that some elements may lift the toxic effect of others, as mentioned above. The induction of phenolic synthesis may be of particular importance in the response of plants to mixed pollutants present in the environment, where an increase in the content of one element is often accompanied by an increase in the content of another element.

## Conclusions

While the results found in this study, indicating a stronger inhibition of plant growth (mainly leaves) during their exposure to 2 elements than 1, do not seem to be a surprise, significant changes in the root structure are not. The study of the role of mutual interactions between Cu, Pb and Zn also showed that (i) the higher content of major elements in individual parts of plants exposed to two metals could result from differences in transport speed; (ii) the appearance of major elements in the substrate in certain ratios may result in a specific response of trees (a specifically browning of leaf edges), and (iii) the higher toxicity of Pb and Zn present simultaneously in Knop solution for leaves than Cu and Pb or Cu and Zn, irrespective of the mutual ratio of the concentrations of these elements.

However, it should be remembered that the efficiency of metal accumulation in roots may reflect the mutual relations between the elements in the culture medium (soil). At the same time, in shoots and the leaves, such an assessment may be burdened with a considerable error due to the different translocation of metals. For this reason, it is justified to assess the role of mutual interactions on the accumulation of elements in the entire biomass of the plant.

## Data Availability

Not applicable.
